# Chronic Stimulation of the Tone of Endogenous Anandamide Reduces Cue- and Stress-Induced Relapse in Rats

**DOI:** 10.1093/ijnp/pyu025

**Published:** 2014-12-19

**Authors:** Claudia Chauvet, Céline Nicolas, Nathalie Thiriet, MD; Virginie Lardeux, Andrea Duranti, Marcello Solinas

**Affiliations:** University of Poitiers, France (Drs Chauvet, Nicolas, Thiriet, Lardeux, and Solinas); INSERM, Experimental and Clinical Neurosciences Laboratory, Poitiers, FRANCE (Drs Nicolas, Thiriet, Lardeux, and Solinas); Dipartimento di Scienze Biomolecolari, Università degli Studi di Urbino “Carlo Bo,”Urbino, Italy (Dr Duranti).

**Keywords:** abstinence, addiction, chronic treatment, endocannabinoid, FAAH, psychostimulants, relapse, stress

## Abstract

**Background:**

The endogenous cannabinoid system plays an important role in motivation, stress, and drug abuse. Pharmacologically, the endocannabinoid system can be stimulated by either agonists of CB1 receptors or inhibition of metabolic degradation of endogenous cannabinoids and consequent increases in their brain levels.

**Methods:**

Here, we investigated whether chronic administration during a period of withdrawal of the fatty acid amide hydrolase inhibitor URB597, which increases anandamide levels, would decrease the risks of relapse to cocaine seeking. Rats were allowed to self-administer cocaine and then they underwent forced withdrawal for 28 days, during which they were treated with URB597 or vehicle. One day after the last injection, we investigated cocaine seeking in one 6h extinction session and relapse triggered by re-exposure to drug-associated cues or a pharmacological stressor.

**Results:**

We found that administration of URB597 significantly decreases cocaine-seeking behavior and cue- and stress-induced relapse.Conclusion: These results suggest that stimulation of the endocannabinoid system could be helpful to prevent relapse to cocaine addiction.

Drug addiction is a chronic relapsing disease that represents a major social and economic burden to our society. One of the most troubling hallmarks of addiction is the long-lasting risk of relapse even after protracted periods of abstinence. Despite the fact that in the last decades great progress has been made in the understanding of the brain mechanisms underlying relapse (Koob and Volkow, 2010), effective therapies for cocaine addiction are still very limited.

One reason for the failure to translate preclinical results into clinical practice could be the fact that most studies using reinstatement models have investigated the effects of a single acute administration of a compound on measures of relapse performed a few minutes later (Shaham et al., 2003). This experimental design has allowed researchers to dissect mechanisms responsible for relapse (Shaham et al., 2003) and could be useful in the case of targeted approaches (Spanagel and Vengeliene, 2013), in which patients are instructed to take the medication when they foresee situations that can trigger relapse. However, this approach does not mimic typical human pharmacological therapies, in which medications are given chronically for several weeks or months and in which events triggering craving and relapse can occur at any time between consecutive administrations of the medication. Importantly, chronic use administration of a pharmacological agent can lead to development of tolerance and the appearance of adverse affects that reduce the clinical efficacy of a medication and patient compliance. Finally, chronic use may be necessary for neuroadaptations to occur and for beneficial effects to kick in, as has been clearly shown with most anti-depressants (Hyman and Nestler, 1996). This could be very important for addiction, which is believed to be at least in part due to drug-induced long-lasting neuroadaptations that result in dysregulation of several brain processes such as memory, salience attribution, emotion, etc. Therefore, in order to cure the disease rather than treating the symptoms, it may be necessary to re-establish a pre-existent equilibrium between circuits that is likely to require chronic administration of therapeutic agents. Unfortunately, only a few studies have investigated the effects of repeated or chronic administration of potential medication drugs (for example, see Baker et al., 2001; Vengeliene et al., 2010; Theberge et al., 2013).

The endocannabinoid system is an endogenous neuromodulatory system comprised of cannabinoid receptors, endogenous ligands, and several protein systems for the synthesis, transport, storage, and degradation of these compounds (Solinas et al., 2008; Mechoulam and Parker, 2013; Piomelli, 2014). Accumulating evidence suggests that the endocannabinoid system plays an important role in a broad range of physiological functions as well as in psychiatric disorders including drug addiction (Solinas et al., 2008; Mechoulam and Parker, 2013; Piomelli, 2014).

Fatty acid amide hydrolase (FAAH) is the most important degradative enzyme for anandamide, and drugs that inhibit these enzymes increase brain and peripheral levels of anandamide (Kathuria et al., 2003). Administration of URB597 has been shown to have positive effects against anxiety (Kathuria et al., 2003), depression (Gobbi et al., 2005; Bortolato et al., 2007), and nicotine addiction-related behaviors (Scherma et al., 2008) without producing reinforcing effects by themselves (Solinas et al., 2006; Justinova et al., 2008). On the other hand, acute administration of URB597 does not alter active self-administration of alcohol (Cippitelli et al., 2008), heroin (Solinas et al., 2005), or cocaine (Justinova et al., 2008; Adamczyk et al., 2009) and reduces relapse to nicotine (Scherma et al., 2008) and cocaine (Adamczyk et al., 2009), but not alcohol (Cippitelli et al., 2008). In addition, acute administration of URB597 decreases some adverse effects associated with withdrawal from drugs such as nicotine (Cippitelli et al., 2011). However, whether chronic exposure to URB597 during a long withdrawal period could reduce cocaine seeking in rat models of relapse has not been studied.

## Methods

### Subjects

Adult (10–11 weeks of age) male Sprague-Dawley rats, experimentally naive at the start of the study, were housed in a temperature- and humidity-controlled room and maintained on a 12 hour light/dark cycle (light on at 7:00 AM). All experiments were conducted during the light phase in accordance with European Union regulations for animal use in research (2010/63/EU).

### General Experimental Design

After 10 days of cocaine or saline self-administration, rats were pseudo-randomly divided into two groups, assuring similar levels of cocaine or saline exposure, and remained abstinent in the animal facility for 28 days. During this period, one group was administered daily with URB597 (0.3mg/kg) between 10:00 and 12:00 AM, whereas the other group received vehicle injection. The day after the last URB597 or vehicle injections, rats were tested for measures of relapse according to a between-within procedure in which seeking during extinction and reinstatement are investigated on the same day (Shaham et al., 2003). For cue-induced reinstatement experiments (Experiment 1), we had a total of four groups: SAL-VEH (n = 5), SAL-URB (n = 5), COC-VEH (n = 9), and COC-URB (n = 10). For stress-induced reinstatement (Experiment 2), we had two groups: COC-VEH (f = 8) and COC-URB (n = 7).

### Cocaine Self-Administration Apparatus and Procedure

Cue-induced reinstatement experiments (Experiment 1) were performed in Imetronic experimental chambers equipped with nose-pokes as operanda (Imetronic, www.imetronic.com). Because stress-induced reinstatement of drug-seeking is more easily obtained using lever-press responding (Chauvet et al., 2009), stress-induced reinstatement experiments (Experiment 1) were performed in Coulbourn experimental chambers equipped with levers as operanda (Coulbourn Instruments, www.coulbourn.com). Ten 6h cocaine self-administration sessions were conducted using a Fixed-Ratio 1 schedule of reinforcement, as previously described (Chauvet et al., 2009). Control “yoked” rats in experiment 1 received an injection of saline each time the paired “master” rat self-administered an injection of cocaine. Active and inactive responses by yoked rats were recorded but had no programmed consequences.

Cocaine-seeking behavior was measured in one 6h extinction session in which cocaine was not delivered. This session was divided into six 1h extinction segments separated by 5min intervals. For cue-induced reinstatement experiments, during this session drug-associated stimuli were not delivered after active responses. A single 1h cue-induced reinstatement session was conducted immediately after the extinction session, during which the presentation of conditioned stimuli (light and pump noise) was made contingent on active nose-pokes. For stress-induced reinstatement experiments, during extinction cocaine-associated cues were present and saline (1ml/kg i.p.) was injected immediately before the 6th hour of the extinction session to allow adaptation to stress effects due to the injection. Then, immediately before the stress-induced reinstatement test session, rats were injected with yohimbine (1.25mg/kg i.p) in accordance to our previous study (Chauvet et al., 2009). The number of active responses was used as a measure of cocaine seeking.

### Drugs

Cocaine HCl was obtained from Cooper (Cooperation Pharmaceutique Francaise, www.cooper.fr) and dissolved in sterile saline solutions (0.9%). Yohimbine HCl (Sigma, www.sigmaaldrich.com) was dissolved in sterile water. The FAAH inhibitor URB597 (synthesized at the University of Urbino Carlo Bo), was dissolved in 5% DMSO (Sigma), 5% Tween-80 (Sigma), and 90% sterile saline. The dose of 0.3mg/kg of URB597 was chosen based on previous results, which demonstrated that chronic treatment with this dose was effective to reduce depression-like behavior, produced no adverse or toxic effects, and increased brain levels of anandamide (Bortolato et al., 2007).

### Statistical Analysis

Differences in drug-seeking behavior were assessed by two-way or three-way ANOVA for repeated measures. Results showing significant overall changes were subjected to a Student-Newman-Keuls post hoc test. Differences were considered significant when *p* < 0.05.

## Results

### Cocaine Self-Administration Training

For both Experiments 1 and 2, rats that were allowed to self-administer cocaine rapidly acquired self-administration behavior and showed a clear preference for the active over the inactive operanda (Figure S1 and S2). In contrast, yoked saline rats (Experiment 1) produced very few responses on both the active and inactive nose-pokes. Importantly, rats were assigned to URB597 or vehicle treatment at the end of the last cocaine self-administration session, assuring that the two groups had similar basal levels of cocaine self-administration. As a consequence of this design, the number of active and inactive responses and the number of cocaine injections did not differ between future URB597 and future vehicle groups (Figure S1 and S2).

### Experiment 1: Effects of URB597 on Cue-Induced Reinstatement

**Figure 1.**
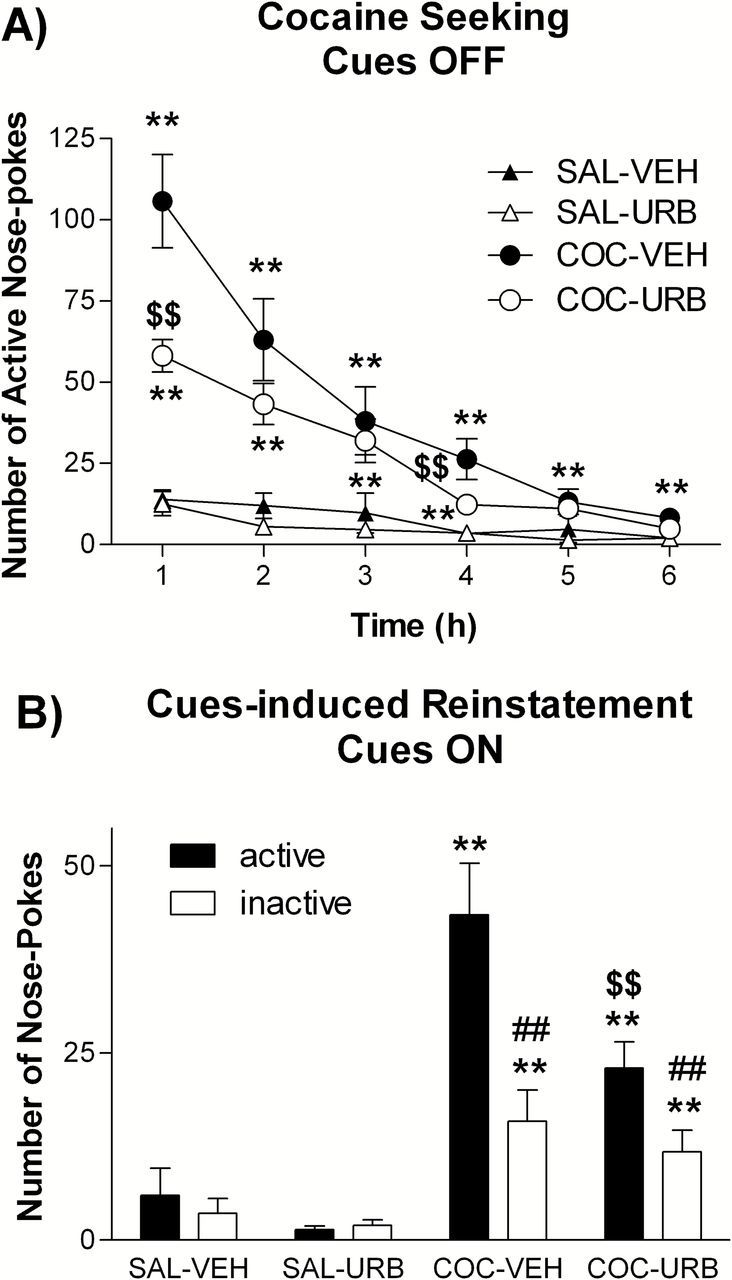
Experiment 1: effects of chronic URB597 treatment on cue-induced reinstatement. (A) Cocaine seeking in a 6h extinction session without cocaine-paired cues and (B) cue-induced reinstatement with reintroduction of the cues in rats administered daily with URB597 (0.3mg/kg i.p.) or vehicle during a 28-day period of abstinence. Note that for reasons of clarity inactive nose-poke responses in A are not shown. Three-way ANOVA followed by Student-Neuman-Keuls post hoc test, **p < 0.01 master cocaine different from yoked saline control, $$*p* < 0.01 URB-treated different from vehicle-treated control, ##*p* < 0.01 active different from inactive nose-pokes.

In extinction sessions performed in the absence of conditioned cues (lights and pump noise), drug-seeking behavior in both URB597 and vehicle rats extinguished within the 6h session (Figure 1A). Importantly, drug seeking, measured as number of active nose-pokes, was significantly lower in URB597 compared to vehicle rats (Figure 1A). As expected, yoked-saline rats produced very few responses (Figure 1A). Inactive responses were higher in master cocaine rats compared to yoked-saline control, but did not differ between URB597- and vehicle-treated rats (data not shown). Statistical analysis revealed a significant effect of drug (cocaine versus saline, F1,25 = 30.39, *p* < 0.0001), of time in the session (F5,25 = 36.20, *p* < 0.0001), a drug x time in the session interaction (F5,25 = 19.71, *p* < 0.0001), a treatment (vehicle vs URB597) x time in the session interaction (F5,25 = 2.47, *p* < 0.05), and a drug x treatment x time in the session interaction (F5,25 = 2.49, *p* < 0.05).

In reinstatement sessions, when presentation of conditioned cues was made contingent on active nose-poking, both URB597 and vehicle rats reinstated cocaine seeking, showing a clear preference for the active over the inactive lever. However, cue-induced reinstatement was significantly lower in URB597 compared to vehicle rats (Figure 1B). Again, yoked-saline rats produced very few responses in this reinstatement session. The number of inactive nose-pokes was higher in cocaine rats compared to saline rats but it did not differ between vehicle- and URB597-treated rats. Statistical analysis revealed a significant effect of drug (F1,25 = 28.10, *p* < 0.0001), of treatment (F1,25 = 4.20, *p* < 0.01), of active device (F1,25 = 16.00, *p* < 0.001), and a drug x active device interaction (F1,25 = 13.41, *p* < 0.05).

### Experiment 2: Effects of URB597 on Stress-Induced Reinstatement

**Figure 2.**
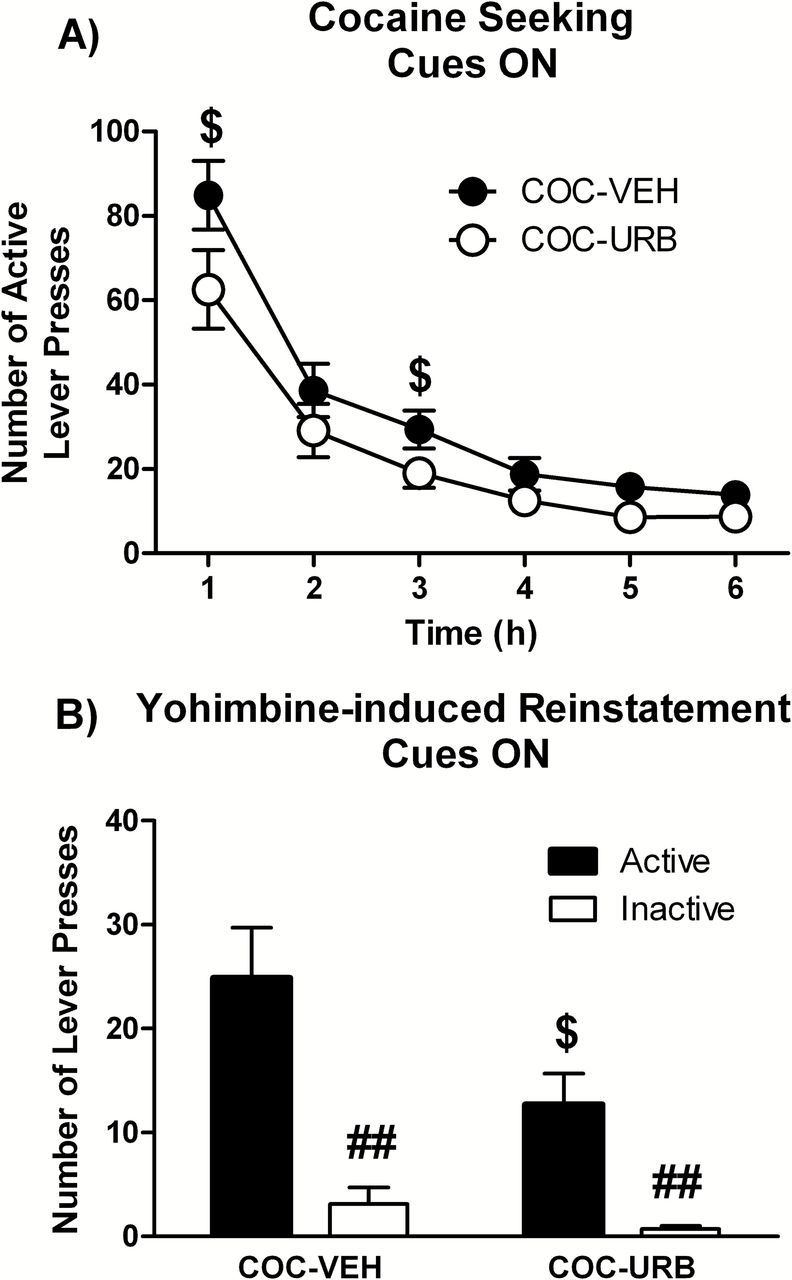
Experiment 2: effects of chronic URB597 treatment on stress-induced reinstatement. (A) Cocaine seeking in a 6h extinction session in the presence of cues previously paired with cocaine and (B) stress-induced reinstatement triggered by the pharmacological stressor yohimbine (1.25mg/kg i.p.) in rats administered daily with URB597 (0.3mg/kg i.p.) or vehicle during a 28-day period of abstinence. Two-way ANOVA followed by Student-Neuman-Keuls post hoc test: $*p* < 0.05 URB-treated different from vehicle-treated control, ##*p* < 0.01 active different from inactive nose-pokes.

Even in an extinction session performed in the presence of conditioned cues, drug-seeking behavior in both URB597 and vehicle rats extinguished within the 6h session (Figure 2A). Importantly, drug seeking, measured as number of active lever presses, was significantly lower in URB597 compared to vehicle rats (Figure 2A). Inactive responses did not differ between URB597- and vehicle-treated rats (data not shown). Statistical analysis revealed a significant effect of treatment (vehicle versus URB597, F1,78 = 11.68, *p* < 0.0001), and of time in the session (F5,78 = 42.97, *p* < 0.0001).

In reinstatement sessions, the pharmacological stressor yohimbine (1.25mg/kg i.p) increased cocaine seeking in both URB597 and vehicle rats, with a clear preference for the active over the inactive lever. However, stress-induced reinstatement was significantly lower in URB597 compared to vehicle rats (Figure 2B). The number of inactive lever presses did not differ between vehicle- and URB597-treated rats. Statistical analysis revealed a significant effect of drug of treatment (F1,26 = 4.20, *p* < 0.01) and of active device (F1,26 = 30.20, *p* < 0.0001).

## Discussion

In this study, we demonstrated that chronic administration of the FAAH inhibitor URB597 during abstinence significantly reduces cocaine-seeking behavior and cue- and stress-induced reinstatement in an animal model of relapse. These results suggest that this class of compounds could represent novel medications for the treatment of cocaine addiction.

### Chronic Treatment with FAAH Inhibitors

Whereas a number of molecules have been proposed in the last decades for the treatment of cocaine addiction, the success of these molecules in clinical settings has been very limited. One of the reasons for the limited success in translating preclinical findings to humans may lay in the fact that the effects of most molecules are tested acutely and the effects of chronic administration, that better mimic human use, are not tested. Whereas a previous study showed that acute administration of URB597 30 minutes before a test session could reduce cocaine- and cue-induced reinstatement (Adamczyk et al., 2009), it was important to investigate whether chronic administration of this compound would be effective in reducing drug seeking in animal models of relapse. In this study we used a chronic treatment with low doses of URB597 during long periods of abstinence and we stopped the chronic treatment the day before the relapse test. This approach has several major advantages compared to the most common strategies to test new molecular agents for the treatment of relapse: (1) it is likely to mimic better human treatment of addicts; (2) it controls for possible development of tolerance or adverse effects to the treatment; and (3) it avoids possible direct effects of the drug on behavioral measures. Importantly, under these conditions administration of URB597 was very effective in decreasing both cocaine seeking in extinction sessions and cue- and stress-induced reinstatement, suggesting that FAAH inhibitors could be effective in facilitating abstinence and preventing relapse over long periods of time.

### Neurobiological Mechanisms

Both acute (Kathuria et al., 2003) and chronic (Bortolato et al., 2007) administration of URB597 inhibits FAAH enzymes and increases levels of anandamide in the brain, namely in the midbrain, the thalamus, and the striatum. Conversely, it has been shown that a history of cocaine self-administration produces significant changes in the levels of anandamide in several brain areas (Bystrowska et al., 2013). In fact, in rats that were exposed to cocaine and then underwent extinction, anandamide levels were decreased in several brain areas, such as the frontal cortex, nucleus accumbens, and hippocampus (Bystrowska et al., 2013). Therefore, administration of URB597 could normalize these levels and restore normal brain functioning, decreasing the risks of relapse.

Anandamide has been characterized as an endogenous cannabinoid ligand (Mechoulam and Parker, 2013; Piomelli, 2014). Chronic exposure to cocaine has been shown to alter the levels of cannabinoid CB1 receptors in the several brain regions (Adamczyk et al., 2012) and, conversely, in a model of depression chronic treatment with URB597 has been shown to alter the levels of cannabinoid receptors in a manner that suggests normalization of dysregulations induced by chronic mild stress (Bortolato et al., 2007). Therefore, normalization of the functioning of the brain endogenous cannabinoid system is the most likely mechanism responsible for the effects of URB597.

On the other hand, anandamide also produces effects though alternative targets such as activation of vanilloid TRPV1 receptors, activation of PPARalpha receptors, or interaction with gap junctions (Pistis and Melis, 2010; Di Marzo and De Petrocellis, 2012). Indeed, the beneficial effects of URB597 against nicotine addiction appear to be mostly related to the activation of PPARalpha receptors, and the effects of cocaine on the electrophysiological activity of dopamine neurons also appears mediated by these receptors (Pistis and Melis, 2010). In addition, other fatty acid amides could also participate in the effects of URB597. However, Bystrowska et al. (2013) have found that during abstinence from voluntary cocaine self-administration, oleoyl-ethanolamine and palmitoyl-ethanolamine are increased in the brain and, therefore, it is unlikely that further increases in these levels could have a therapeutic beneficial effect. Further research is needed to fully characterize the mechanisms responsible for the effects of FAAH inhibitors and to determine whether systems other than the endocannabinoid system participate in the effects of URB597.

### Conclusions

The present data demonstrate that administration of the FAAH inhibitor URB597 reduces cocaine-seeking and cue- and stress-induced reinstatement. Therefore, drugs that increase the levels of endogenous anandamide and stimulate the endogenous cannabinoid system may be useful pharmacological tools for the treatment of cocaine addiction.

## Supplementary Material

For supplementary material accompanying this paper, visit http://www.ijnp.oxfordjournals.org/

## Statement of Interest

None.

## Supplementary Material

Figure S1
