# Supplementary material for: Chronic Stimulation of the Tone of Endogenous Anandamide Reduces Cue- and Stress-Induced Relapse in Rats
Source: Int J Neuropsychopharmacol. 2014 Dec 19;18(1):pyu025. doi: 10.1093/ijnp/pyu025 (PMC4368869; doi:10.1093/ijnp/pyu025)
Supplement: Figure S1 [file ijnp_pyu025_index.html]

Supplementary Data | International Journal of Neuropsychopharmacology

## Supplementary Data

Data files

**Files in this Data Supplement:**

- Supplementary Data - Supplementary Data
- Supplementary Data - Supplementary Data
- Supplementary Data - Supplementary Data
