# Supplementary material for: Chronic Stimulation of the Tone of Endogenous Anandamide Reduces Cue- and Stress-Induced Relapse in Rats
Source: Int J Neuropsychopharmacol. 2014 Dec 19;18(1):pyu025. doi: 10.1093/ijnp/pyu025 (PMC4368869; doi:10.1093/ijnp/pyu025)
Supplement: Figure S1 [file URB_Coc_Supplementary_Figure_Legends.docx]

Supplementary Figure Legends

**Figure S1. Experiment 1: Cocaine self-administration training before forced abstinence and treatment with URB597.** Master cocaine rats (COC-VEH and COC-URB) were allowed to self-administer cocaine for ten 6-h sessions while yoked saline rats (SAL-VEH and SAL-URB) received passively saline injections. At the end of the self-administration training, rats were assigned to URB597 or vehicle treatment and underwent a 28-day period of forced abstinence. Two-Way ANOVA followed by Student-Neuman-Keuls post-hoc test, **, P < 0.01 different from yoked saline control.

**Figure S2. Experiment 2: Cocaine self-administration training before forced abstinence and treatment with URB597.** In these experiment, all rats were allowed to self-administer cocaine for ten 6-h sessions. At the end of the self-administration training, rats were assigned to URB597 or vehicle treatment and underwent a 28-day period of forced abstinence.
